# Identification, Characterization, and Evaluation of Nematophagous Fungal Species of *Arthrobotrys* and *Tolypocladium* for the Management of *Meloidogyne incognita*

**DOI:** 10.3389/fmicb.2021.790223

**Published:** 2021-12-10

**Authors:** Rami Kassam, Jyoti Yadav, Gautam Chawla, Aditi Kundu, Alkesh Hada, Nisha Jaiswal, Haritha Bollinedi, Deeba Kamil, Prameela Devi, Uma Rao

**Affiliations:** ^1^Division of Nematology, ICAR-Indian Agricultural Research Institute, New Delhi, India; ^2^Division of Agricultural Chemicals, ICAR-Indian Agricultural Research Institute, New Delhi, India; ^3^Division of Genetics, ICAR-Indian Agricultural Research Institute, New Delhi, India; ^4^Division of Plant Pathology, ICAR-Indian Agricultural Research Institute, New Delhi, India

**Keywords:** *Arthrobotrys thaumasia*, *Tolypocladium cylindrosporum*, nematophagous fungi, *Meloidogyne incognita*, biocontrol, parasitism, metabolite profiling

## Abstract

Root-knot nematodes belonging to the genus *Meloidogyne* are agriculturally important pests, and biocontrol strategies offer safer alternatives for their management. In the present study, two fungal species from Indian soils were identified as *Arthrobotrys thaumasia* and *Tolypocladium cylindrosporum* based on morphological characteristics and further confirmed using molecular markers. *In vitro* evaluation of *A. thaumasia* against *M. incognita* and *Caenorhabditis elegans* showed 82 and 73% parasitism, respectively, whereas *T. cylindrosporum* gave 65.2 and 57.7% parasitism, respectively. Similarly, culture filtrates of *A. thaumasia* caused 57.7 and 53.7% mortality of *M. incognita* and *C. elegans*, respectively, whereas *T. cylindrosporum* caused higher mortality of 87.3 and 64%, respectively. Besides, greenhouse evaluation of both fungi against *M. incognita* infecting tomato significantly reduced nematode disease burden reflecting parasitic success measured as the total number of galls, egg masses, eggs per egg mass, and derived nematode multiplication factor. Application of *A. thaumasia* and *T. cylindrosporum* reduced nematode multiplication factor by 80 and 95%, respectively, compared with control. General metabolite profiling of tested fungi using gas chromatography–mass spectrometry and ultra-performance liquid chromatography–quadrupole/time of flight mass spectrometry reported for the first time here showed presence of various volatile and non-volatile compounds with nematicidal activity, *viz.*, trimethyl-heptadiene, methyl-hexadecanol, dodecadienal, decane, terpendole E, dodecane, acetamido-6-anthraquinone, and hexadecanol. Also, other compounds such as undecane, dibutyl-disulfide, octadecenal, paganin, talathermophilin, dactylarin, tolypyridone A, tolypyridone B, pyridoxatin, and destruxin were identified, reported in the literature to possess antibacterial, antifungal, and insecticidal properties. This is the first report of the occurrence of both fungi from India and pioneer demonstration of *T. cylindrosporum* for root-knot nematode management.

## Introduction

Plant-parasitic nematodes (PPNs) are considered hidden enemies and pose a major threat to both agricultural and horticultural crops. They have a universal distribution and cause an estimated yield loss of US$ 173 billion every year (Elling, 2013). Amongst the top 10 economically important PPN species worldwide, root-knot nematodes (RKNs) belonging to the genus *Meloidogyne* are considered to be the most severe ones (Rady, 2018). The pre-parasitic J2s of the most important RKN species, *Meloidogyne incognita*, penetrate and infect the root tip using a hollow needle-like protrusible stylet. The stylet is used for probing the plant tissue and entering into the vascular cylinder, where it injects the esophageal gland secretions that induce the formation of specialized feeding cells known as giant cells by suppressing the plant host immune system. Eventually, the feeding J2s undergo consecutive molts to J3 (third stage juveniles) and J4 (fourth stage juveniles) stages and young females that develop into reproducing mature females that lay eggs (Poveda et al., 2020). Despite enormous damage caused by PPNs in various crops, there is still a dearth of effective and efficacious nematode management option(s).

Traditionally, management of PPNs relied on integrated cultural and physical tactics such as clean cultivation practices, crop rotation, solarization of the soil before planting, adequate fertilization, and removal of infected plants and weeds (Oka et al., 2007). Additionally, one of the most effective, economical, and environmentally safe methods to reduce yield losses from nematode diseases is to use nematode-resistant cultivars. However, commercially viable resistant varieties and/or cultivars may not be either available for all the crops or limited in numbers for only specific crops due to lack of resistant sources required for varietal development (Ploeg et al., 2019). Chemical nematicides are the mainstay due to their ability to reduce high densities of nematodes in the soil in a short period to avoid significant yield losses (Regmi and Desaeger, 2020). However, due to their high toxicity and possible environmental and health hazards, most of the chemical nematicides, fumigants, and insecticides have been withdrawn or banned from the global market (Ruiz-Suárez et al., 2015; Banakar et al., 2020; Hada et al., 2021b,c). In addition, limited label claim of some of the recently introduced synthetic chemicals such as fluensulfone, fluopyram, etc., restricts their usage in all the crops and also against different nematodes. Therefore, using novel comprehensive approaches is the need of the hour for sustainable agricultural production.

Nematode management using bio-control strategies has been known to be a safer alternative and practical approach. This is reflected by a considerable investment of venture capital in research required for developing biocontrol options (Escudero et al., 2017). One of the biocontrol approaches is to regulate nematode populations using nematophagous fungi, which have antagonistic activity against infective juveniles (Tian et al., 2007). Nematophagous fungi and/or endophytic fungi can directly attack, kill, immobilize, or repel nematodes, confuse them while finding their host, interfere with giant cell development, compete for resources, or use a combination of these options (Schouten, 2016). It can also capture, parasitize, or paralyze nematodes and act as natural enemies of plant and animal-parasitic nematodes. They are divided into four groups, i.e., endoparasitic fungi, nematode-trapping fungi (NTF), opportunistic fungi, and toxin-producing fungi (Siddiqui and Shaukat, 2004). NTFs are the unique group of soil-inhabiting fungi that can switch from the saprophytic to pathogenic lifestyle once they come in contact with nematodes as a response to nutrient depletion, and the predatory behavior adapted by them is exciting. *Arthrobotrys oligospora* Fres. 1852 (Orbiliaceae: Orbiliales) was the first recognized nematode-trapping fungus and the most abundant in the environment (Niu and Zhang, 2011). The nematode-trapping process of the network structure of *A. oligospora* demonstrated that a specialized mycelial structure traps the nematodes followed by penetration of nematode cuticle, after which it digests the body contents (Barron, 2003). Zhang et al. (2010) revealed that *A. scaphoides* isolated from soil using *Panagrellus redivivus* nematodes as bait caused three-dimensional (3D) adhesive networks that trapped nematodes within 2 days. The utilization of NTFs will be an attractive alternative for the biological control of infective larvae. The importance of physicochemical processes of NTF is of immense interest, and researchers have collectively revealed that trapping and/or immobilization are associated with upregulation of several signaling pathways, intercellular communications, production of adhesive proteins, and organic metabolites as well as nitrate assimilation (Yang et al., 2011).

Besides, Wang B.L. et al. (2018) highlighted that *Caenorhabditis elegans* was attracted toward *A. oligospora* due to three fungal metabolites, namely 2(5*H*)-furanone, furan-2-yl methanol, and furan-2-carbaldehyde. However, the compound 3-hydroxy-2-methyl-4*H*-pyran-4-one (known as maltol) displayed a significant increase in the formation of 3D traps. Likewise, a quantity of four fungal metabolites, e.g., desferriferrichrome, linoleyl alcohol, non-adecanamide, and citicoline, were found to increase when fungi switch the lifestyle to the predatory stage, and they also showed considerable nematicidal activity (Kuo et al., 2020). Metabolite profiling of 100 wild isolates of NTF in three different species, *A. oligospora*, *Arthrobotrys thaumasia*, and *Arthrobotrys musiformis*, revealed the production of thousands of metabolites belonging to various structural families such as peptide, siderophore, fatty alcohol, and fatty acid amide during their interaction with *C. elegans* as demonstrated by liquid chromatography–mass spectrometry (LC–MS) analyses.

The endophytic entomopathogenic fungus *Tolypocladium* spp. is known to survive in the soil during the absence of insects using nematodes as alternate hosts (Samson and Soares, 1984). *T. cylindrosporum* is a saprotroph and an entomopathogenic fungus studied as a biological control agent against insects of several orders but, to the best of our knowledge, so far, not known to be useful against PPNs. Nematophagous fungi serve as both predators and decomposers in the environment, and there might be regional differences in the effectiveness of different fungal isolates (Wang F.H. et al., 2017). Hence, isolation, identification, and characterization of native strains of fungi with predatory activity are crucial to identify the potential fungi along with an understanding of their ecology, biology, mode of action, and interactions to exploit them successfully against target PPNs.

In view of the importance of nematophagous fungi as biocontrol agents for nematode management, we had isolated and identified 81 fungal isolates up to generic level from 17 soil samples collected across different states of India using *C. elegans* and *M. incognita* as bait (Kassam et al., 2021). In continuation, the present study is aimed to identify the species of two important fungal isolates, *Arthrobotrys* and *Tolypocladium*, using morphological characters and molecular markers. Furthermore, the effect of nutrition, temperature, and pH on growth rate and trap formation has been studied. Besides, fungal parasitization against *M. incognita* and *C. elegans* was evaluated under *in vitro* and *in vivo* conditions. We have also profiled the volatile and non-volatile chemical compounds [volatile organic compounds (VOCs)] produced by these fungal isolates.

## Materials and Methods

### Nematode Cultures

*C. elegans* strain N95 was maintained on a nematode growth medium with *Escherichia coli* strain OP50 lawn. A chunk of agar containing hundreds of worms was cut and transferred onto an overnight grown OP50 lawn in a fresh Petri plate. Plates were incubated at 25°C for 3 days for nematode multiplication.

The authenticated population of *M. incognita* was maintained and multiplied on tomato roots (*Solanum lycopersicum* L. cv. Pusa Ruby) in the greenhouse. Approximately 35-day-old plants were harvested, roots washed free of soil, and used for collecting fresh egg masses, which were hatched *via* modified Baermann’s technique (Whitehead and Hemming, 1965) to obtain infective second-stage juveniles (J2s) required for all the experiments.

### Morphological and Molecular Classification of the Fungal Isolates

In the present study, we have taken the two Indian isolates of *Arthrobotrys* and *Tolypocladium* for species identification. Pure cultures of both the fungal isolates were grown separately in potato dextrose broth (PDB) at 25°C for 1 week. Cultures of *Arthrobotrys* spp. and *Tolypocladium* spp. were observed under an Olympus BX50 compound microscope, and morphological measurements of conidia, conidiophores, and phialides (length and width) were recorded. For molecular characterization, genomic DNA was extracted from a 1-week-old mycelial mat using the CTAB method as previously described by Wang et al. (2015). Polymerase chain reaction (PCR) analyses were carried out with genomic DNA extracted from the fungal isolates to amplify the two markers; internal transcribed spacers (ITSs) and β-tubulin using universal primers, ITS-1 (5′-TCCGTAGGTGAACCTGCGG-3′) and ITS-4 (5′-TCCTCCGCTTATTGATATGC-3′) as well as βt2A (5′-GGTAACCAAATCGGTGCTGCTTTC-3′) and βt2B (5′-ACCCTCAGTGTAGTGACCCTTGGC-3′), respectively. PCR amplification was carried out as per the procedure by Glass and Donaldson (1995). The amplified products were analyzed by electrophoresis on a 1.2% (w:v) agarose gel (Sigma Aldrich, United States) and visualized using a gel documentation system (Alpha Image Analyzer, United States). PCR products were further purified using a PCR clean-up kit (Macherey-Nagel, Germany) and sequenced (Applied Biosystems, United States). The sequences of ITS and β-tubulin generated for the tested fungi were compared with previously reported sequences in the GenBank database. All the sequences were imported for alignment by setting default parameters in the ClustalW algorithm into a MEGA X software application package. To search for homologs of both genes, sequences from each isolate were subjected to the Nucleotide Basic Local Algorithm Search Tool of the National Center for Biotechnology Information.^1^ The sequences were further analyzed and submitted to GenBank. Additionally, phylogenetic trees using the sequences of ITS and β-tubulin of the tested isolates were constructed in MEGA X using the maximum composite likelihood approach considering 1,000-bootstrap replications under Kimura two-parameter distance models (Kimura, 1980). For this, *Neurospora crassa* and *Cordyceps ophioglossoides* were used as out-groups (Sung et al., 2007; Swe et al., 2008; Wright et al., 2009). In addition, the identified fungal isolates were submitted to the Indian Type Culture Collection (ITCC), which is an affiliate member of the World Federation for Culture Collections and is registered with the World Data Centre for Microorganisms (registration number 430).

### Effect of Nutrition, Temperatures, and pH on Growth Rate and Trap Formation of the Selected Fungi

Pure cultures of both the fungi were grown in six different media, *viz.*, potato dextrose agar (PDA), cornmeal agar (CMA), Czapek malt agar (CzMA), rose bengal agar (RBA), peptone-yeast-glucose, and synthetischer nährstoffarmer agar (SNA) (HiMedia, India) in 90-mm diameter (dm) Petri plates at 25°C to examine the differences in structure, color, growth rate, and sporulation among colonies. For this, a small piece of agar measuring around 5 × 5 mm was cut from a well-established colony and placed upside down at the center of the fresh Petri plate containing different media. Three replicates (*n* = 3) for each medium were used, and observations were made for all the replicates separately. The fungal growth was determined at 3-, 6-, 9-, and 12-day post-inoculation (dpi) by measuring the colony diameter. The growth rate was estimated as growth rate per day (mm/day) = [the average diameter of hyphae measured in that day - 5 (diameter of original inoculum)]/days of culture, as described earlier by Wang F.H. et al. (2017).

Furthermore, the medium that supported the maximum growth rate was used to evaluate the effect of different temperatures, *viz*., 15, 20, 25, 30, and 35°C, by following the procedures described earlier. Additionally, a combination of the specific medium and temperature supporting maximum fungal growth was studied to evaluate the effect of different pH levels (ranging from 4 to 10). Three replicates (*n* = 3) were used for each treatment. The growth rate was estimated at 3, 6, 9, and 12 days after inoculation by a formula adopted from Wang F.H. et al. (2017). The mean value of the diameter of fungal hyphae was measured each day in each group and used for calculating the growth rate by deducting the original diameter of the fungal disc used for sub-culturing.

### *In vitro* Efficacy of Fungal Filtrate on Nematode Mortality

The two selected fungi were grown on a PDB medium for 10 days at 25°C with 180 rpm in an incubator shaker. Approximately 100 surface-sterilized nematodes (both *M. incognita* and *C. elegans*) were added “separately” into 5-ml Eppendorf tubes containing 3-ml fungal filtrate (FF) of each isolate. The tubes were incubated on the shaker at 120 rpm at 25°C for 3 days. The worms were washed thrice with sterile water (SW) followed by re-incubation in SW for 24 h at 25°C for revival in the nematode movement. Then, the nematodes were examined under a stereo binocular microscope to record dead and live nematodes, and nematode mortality percentage was calculated as% mortality = (number of dead nematodes/total number of nematodes) × 100. There were three replicates (*n* = 3) for each treatment, and observations were made for all the replicates independently. Worms in the PDB medium served as the control for comparison.

### *In vitro* Evaluation of Direct Fungal Parasitism Against *M. incognita* and *C. elegans*

Fungal parasitism against *M. incognita* J2s and *C. elegans* L3s was evaluated in water agar plates. Briefly, fungi were grown on PDA at 25°C for 6 days, and then, 5-dm discs were transferred into 2% water-agar plates containing 1% ampicillin (100 μg ml^–1^). The plates were incubated at 25°C for 3 days, and then 100 surface-sterilized worms were added to the plates separately (Contina et al., 2017). The number of captured larvae was scored after 3 days using a light microscope (40×). Three replicates (*n* = 3) were used for each treatment and compared with control (nematodes only in water agar medium). Parasitization was calculated as previously described by Siddiqui and Shaukat (2004) as% parasitization = (number of parasitized nematodes/total number of nematodes) × 100.

Captured nematodes of both *M. incognita* J2s and *C. elegans* L3s were also examined under a scanning electron microscope. For this, worms were fixed in 2% glutaraldehyde in 0.1-M phosphate buffer (pH 7.2) overnight, followed by 2% osmium tetroxide fixation for 6 h and dehydration using a series of ethanol as described by Den Belder et al. (1993).

### *In vivo* Evaluation of Fungi Against *M. incognita*

One-month-old healthy tomato seedlings (*S. lycopersicum* L. cv. Pusa Ruby) were transplanted into 4-inch dm pots filled with 400-g autoclaved soil, sand, and farmyard manure (50:25:25), which were kept in the greenhouse. At the same time, fungal suspensions were prepared by culturing the colonies on medium, which showed a high sporulation level, and incubated at 25°C for 10 days. Subsequently, 5 ml of sterile distilled water was added to the plate and the spores were scraped using a spatula. The mixture was placed in a small beaker and stirred for 10 min, filtered using cheesecloth, and quantified using a hemocytometer on a light microscope. Finally, the fungal suspensions were adjusted to get 1 × 10^6^ spores/ml and used for inoculation around the root zone during transplantation. After 1 week, 800 J2s of *M. incognita* were inoculated at the rate of 2 J2s per gram of soil, and the pots were maintained in the greenhouse. Plants that received sterile distilled water served as control. Five replicates (*n* = 5) were used for each treatment, and observations were made for all the replicates individually. Plants were carefully harvested 45 dpi, and roots were washed free of soil. Plant growth parameters, *viz*., plant length, fresh weight, and dry weight were recorded. Additionally, nematode disease burden was determined as the number of galls, egg masses, and eggs per egg mass and used for deriving the nematode multiplication factor (MF) as [(total number of egg masses per plant × average number of eggs per egg mass) ÷ initial nematode inoculum level] as described previously by Hada et al. (2020, 2021a).

### Characterization of Volatile and Non-volatile Molecules of the Selected Isolates

#### Extraction of Fungal Volatiles for Gas Chromatography–Mass Spectrometry Analysis

The selected fungal isolates were grown on PDA media (HiMedia, India) in Petri plates (90 mm diameter) for 10 days. Fungal mats along with media were taken out separately and extracted with hexane (50 ml × 3) thrice using a bath sonicator at 30-Hz amplitude for 30 min. The extracts were filtered, pooled, and passed through anhydrous sodium sulfate (20 g) to remove traces of water if any. The extracts were concentrated separately under reduced pressure in a rotary evaporator (Heidolph, Germany) and dissolved in gas chromatography–mass spectrometry (GC–MS) grade hexane for further analysis (Saha et al., 2015a).

#### Analysis by Gas Chromatography–Mass Spectrometry

GC–MS analysis was carried out using 7890A GC (Agilent Technologies, United States) equipped with an HP-5MS column (30 m × 0.25 mm/0.25 μm, Agilent Co., United States), which was directly connected to a triple-axis HED-EM 5975C mass spectrometer (Agilent Co., United States). The injection volume was 1 μl with flow mode in split control. The carrier gas flow was set at 1 ml min^–1^ helium. The oven temperature was initially held at 40°C for 2 min. Thereafter, the temperature was raised with a gradient of 3°C min^–1^ until the temperature reached 130°C and held for 2 min. Again, the temperature was raised with a gradient of 5°C min^–1^ up to 220°C and held for 1 min. Finally, the oven temperature was raised to 280°C with an increment of 10°C min^–1^. The total runtime was 59 min. The MS acquisition parameters were set with the ion source temperature 175°C, electron ionization 70 eV, full scan mode (50–550 mass units), and transfer line temperature 250°C. Compounds were identified by matching their mass spectra. Volatile organic compounds (VOCs) were identified by library matching from the National Institute of Standards and Technologies Mass Spectra Library (Saha et al., 2015a).

#### Extraction of Fungal Metabolites for Ultra-Performance Liquid Chromatography– Quadrupole/Time of Flight-Electrospray Ionization–Mass Spectrometry Analysis

The selected fungi were again grown on PDA for 10 days. Fungal mats along with media were taken out separately and extracted with methanol (50 ml, each) thrice using a bath sonicator at 30 Hz. amplitude for 30 min. The extracts were filtered and pooled, and the solvent was evaporated under vacuum in a rotary evaporator that resulted in respective concentrates. The concentrates obtained for each fungus were dissolved in LC–MS grade methanol separately for further analysis (Saha et al., 2015b).

#### Analysis by Ultra-Performance Liquid Chromatography–Quadrupole/Time of Flight-Electrospray Ionization–Mass Spectrometry

The analysis was performed on ultra-performance liquid chromatography–quadrupole/time of flight mass spectrometry (QToF-MS, Synapt G2 HDMS, Waters Corporation, Manchester, United Kingdom). The QToF-MS was operated with electrospray ionization (ESI) at a nominal mass resolution of 20,000 and controlled by MassLynx 4.1 software. The data acquisition was made with the MS^E^function in continuum mode in the range of m/z 50–1,200. The chromatographic separation was performed on an ACQUITY Ultra-Performance Liquid Chromatography (UPLC) BEH C_18_ column (2.1 × 100 mm, 1.8 μm, Waters India Pvt. Ltd., Bangalore) at 35°C. The mobile phase consisted of A phase: methanol–water (20:80) and B phase: methanol–water (80:20) with 0.1% formic acid in both the phases. A gradient program was used with 0.4 ml/min flow rate, with 0–4.0 min 100% A, 4.0–7.0 min 70% A, 7.0–12.0 min 50% A, 12–15 min 30% A, and 15–25.0 min 100% A. The injection volume was 5 μl, and the samples were maintained at 25°C throughout the analysis (Saha et al., 2015b).

#### Statistical Analysis

Data of laboratory experiments were analyzed in a completely randomized design. Greenhouse experiments were conducted in the randomized complete block design, and data were subjected to analysis of variance and Duncan’s multiple range test at 1 and 5% level of significance using statistical package version 160 (SPSS 16.0; IBM Corp., United States). All the experiments were conducted thrice to validate the final outcome.

## Results

### Morphological and Molecular Classification of the Fungal Isolates

**FIGURE 1:**
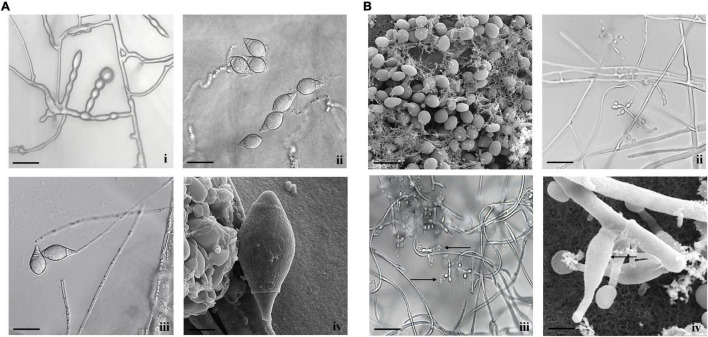
Morphological characteristics of **(A)** *A. thaumasia* At_RK **(i)** chlamydospores, **(ii)** conidia, **(iii)** conidiophores with conidia (scale bar = 20 μm), **(iv)** conidia observed by scanning electron microscope (SEM) (scale bar = 5 μm), **(B)** *T. cylindrosporum* Tc_RK **(i)** conidia observed by SEM (scale bar = 5 μm), **(ii)** conidiophores with phialides (scale bar = 20 μm), **(iii)** conidiophores with phialides along with conidia (scale bar = 20 μm), **(iv)** phialides under SEM (scale bar = 2 μm).

Species identification has been primarily made using morphological characters along with the measurement of taxonomically useful features. The *Arthrobotrys* isolate exhibited straw white-colored colonies with raised concentric rings along with thin and hairy rings on PDA media. The length and width of conidia and conidiophores of *A. thaumasia* were approximately 24.58–60 and 10.15–22.88 μm, as well as 211–446 and 2.3–5.4 μm (*n* = 50), respectively. Furthermore, the shape of the conidium appeared as an inverted pear with 1–3 septa. All these characteristics matched with the reported description of the fungus *A. thaumasia* (Supplementary Table 1 and Figure 1A), and we designated our isolate as *A. thaumasia* At_RK.

Similarly, in the case of *Tolypocladium* isolate, the colony appeared hairy with whitish cream as well as reverse yellow to pale on PDA media. The conidium was 2–4.3 and 1.3–1.7 μm in length and width, respectively. Likewise, the length and width of conidiophores were approximately 31–44 and 1.1–2.8 μm, respectively. The phialide length and width were 4.5–8.5 and 2–3.2 μm, respectively. Finally, conidia hyaline were smooth-walled, short cylindrical, straight and/or slightly curved, and both ends obtusely rounded. Comparison of all these characteristics matched with the reported description of *T. cylindrosporum* (Supplementary Table 1 and Figure 1B), and our isolate was designated as *T. cylindrosporum* Tc_RK.

To further confirm the species identity, the fungi discussed earlier were characterized using two molecular markers, ITS and β-tubulin. Sizes of ITS and β-tubulin that could be amplified in both the isolated fungi were 580 and 370 bp, respectively. Purified PCR products were sequenced and submitted to the National Center for Biotechnology Information database (Supplementary Table 2). Homology search of ITS and β-tubulin sequences using BLAST program showed that *A. thaumasia* At_RK sequence was 99% (accession: KT215216.1) and 97% (accession: EU977531.1) identical to earlier reported sequences of *A. thaumasia*, respectively. Similarly, the ITS sequence of *T. cylindrosporum* Tc_RK was 99.06% (accession: NR_167967.1), identical to a previously reported sequence of *T. cylindrosporum*.

Furthermore, we constructed an evolutionary tree based on the sequences of the ITS, and β-tubulin that demonstrated *A. thaumasia* At_RK was closest to *A. thaumasia* strain CBS 376 97 (accession: KT215216.1), whereas β-tubulin sequence was close to *A. thaumasia* isolate 111 (accession: EU977531). Similarly, the ITS sequence of *T. cylindrosporum* Tc_RK was closest to *T. cylindrosporum* isolate TCDAs18R1A9 (accession: MT911434.1), whereas that of β-tubulin was close to *T. tropicale* strain IQ214 (accession: KF747166.1) and *T. tropicale* strain MX338 (accession: KF747190.1). The results of phylogenetic analysis based on the sequence of ITS and β-tubulin indicated that the isolates in the present study could be different geographical strains of *A. thaumasia* and *T. cylindrosporum* (Supplementary Figure 1).

Additionally, the studied fungal isolates were submitted to ITCC with strain/accession numbers: ITCC8969 for *T. cylindrosporum* Tc_RK and ITCC8970 for *A. thaumasia* At_RK (Supplementary Table 2).

### Effect of Nutrition, Temperatures, and pH on Growth Rate and Trap Formation of the Fungal Colonies

Selected fungal isolates cultured on different nutrient media showed clear variations in the growth rates and trap formation at different time intervals. The highest growth rate of *A. thaumasia* was observed significantly in SNA media (2.00 mm/day), followed by CMA (1.375 mm/d) and CzMA (1.272 mm/day) compared with other tested media after 3 days (Supplementary Figure 2A). Likewise, at 6 dpi, the growth rates were observed to be significantly higher in SNA (1.432 mm/day), CMA (1.405 mm/day), PDA (1.405 mm/day), and CzMA (1.395 mm/day) media, respectively. Finally, the hyphae covered the entire Petri plate, and growth rates of fungus were equal in all the media after 9 dpi (0.94 mm/day) and 12 dpi (0.71 mm/day). Concurrently, sporulation was observed, and the number of spores was counted using a hemocytometer. The maximum sporulation was noticed on RBA (30.3 × 10^4^ spore/ml) followed by CzMA medium (9.1 × 10^4^ spore/ml) (Supplementary Table 3). The response to different media shows that *A. thaumasia* mycelia are very sensitive to salts and amino acids because they promote trap formation at specific nutrient combinations and inhibit it partially or completely at other combinations. There are significant differences among the different media compositions (*p* < 0.01).

In the case of *T. cylindrosporum* isolate, the growth was slow in all the tested media (Supplementary Figure 2B), but PDA media comparatively showed a considerable growth rate (0.64 mm/day) at 3 and 6 dpi. Subsequently, the maximum growth rate was observed in the PDA media (0.525 mm/day) and lowest in CMA media (0.328 mm/d) after 9 and 12 dpi. Consequently, the fungal sporulation was also observed in all the tested media, and the maximum sporulation was obtained on SNA media (9006.3 × 10^4^ spore/ml) followed by PDA (7203.3 × 10^4^ spore/ml) and CMA media (3427.2 × 10^4^ spore/ml) (Supplementary Table 3 and Supplementary Figure 3A).

Furthermore, *A. thaumasia* grown on SNA media that provided a maximum growth rate was used to evaluate the effect of different temperatures. The data indicate that the growth rate of *A. thaumasia* was significantly higher at 30, 25, 20, and 15°C (0.293, 0.634, 0.61, and 0.523 mm/day) after 3, 6, 9, and 12 dpi, respectively. In the case of *T. cylindrosporum* that grew on PDA media, it exhibited the quickest growth rate at 20°C after 3 and 6 dpi (0.525 and 0.465 mm/day, respectively), and the highest growth was noticed after 9 and 12 dpi (0.563 and 0.475 mm/day, respectively) as compared with other treatments (*p* < 0.01). The fungus could not grow at 35°C after 3 and 6 dpi but could grow in the range from 15 to 30°C (Supplementary Figure 3B).

Additionally, a combination of the specific media and temperature supporting maximum fungal growth was studied to evaluate the effect of different pH levels. For this, an isolate of *A. thaumasia* was cultured on SNA media at 30°C. The result showed that the radial growth rate at pH 6–9 (8.575 and 8.475 mm/day) was significantly faster than other tested pH after 3 days. The highest growth under different pH recorded was at pH 9 > pH 6 > pH 7 = pH 8 > pH 10 > pH 5 > pH 4. Similarly, a culture of *T. cylindrosporum* grown on PDA medium at 20°C caused the highest growth rate at pH 6 (1.55–5.15 mm/day) followed by pH 9 and 10, and minimal growth was recorded at pH 4 (0.525–1.975 mm/day) (Supplementary Figure 3C).

### *In vitro* Evaluation of Fungal Filtrate of the Tested Isolates on Nematode Mortality

The FF of the selected isolates were found to be effective against both *M. incognita* J2s and *C. elegans* L3s compared with control in the PDB medium. *T. cylindrosporum* Tc_RK and *A. thaumasia* At_RK caused 87.3 ± 6.02 and 57.7 ± 3.5% mortality of *M. incognita* J2s, respectively. Likewise, FF of *T. cylindrosporum* Tc_RK and *A. thaumasia* At_RK caused 64 ± 3.6 and 53.7 ± 2.3% mortality in *C. elegans* L3s (Supplementary Table 4). Both *M. incognita* and *C. elegans* worms exhibited normal behavior in the PDB control without any mortality.

### *In vitro* Evaluation of Direct Fungal Parasitism Against *M. incognita* and *C. elegans*

**FIGURE 2:**
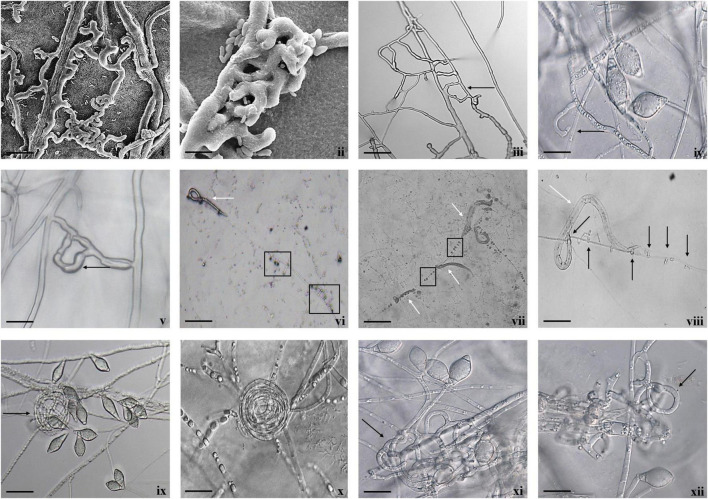
Diversity of trapping structures of *A. thaumasia* At_RK. **(i,ii)** Scanning electron micrographs showing process of trap formation of adhesive two-dimensional (2D) network (scale bar = 20, 5 μm), (**iii**) adhesive 2D (scale bar = 50 μm), **(iv,v)** vegetative hyphae along with an initial branch forming a loop (black arrow) by turning around to meet a peg formed on parent hypha (scale bar = 50, 20 μm), **(vi–viii)** non-constricting rings (black arrows), capturing nematodes (white arrows), **(ix,x)** non-constricting rings (scale bar = 20 μm), **(xi,xii)** 3D adhesive network (scale bar = 20 μm).

**FIGURE 3:**
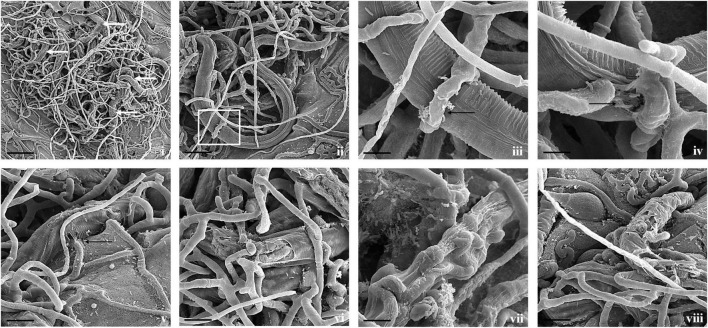
Scanning electron micrographs of interaction between *A. thaumasia* At_RK and *M. incognita*. **(i,ii)** Fungal colonization against many nematodes (white arrows), larvae trapped in fungal networks at various points on nematode body (scale bar = 50, 20 μm), **(iii)** magnification of area highlighted (panel ii) showing pressure caused by modified hyphae on nematode cuticle (scale bar = 5 μm), **(iv)** hyphal penetration site (black arrows) on nematodes cuticle along lateral lines (scale bar = 5 μm), **(v,vi)** fungus rupturing nematode cuticle (scale bar = 10 μm), **(vii,viii)** consumption of nematode body contents by fungus, surface of *M. incognita* is irregular indicating internal growth of hyphae (scale bar = 5, 10 μm).

**FIGURE 4:**
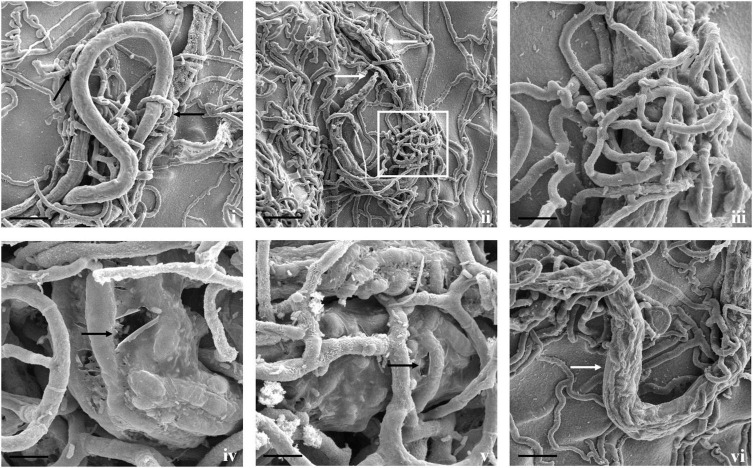
Scanning electron micrographs of interaction between *A. thaumasia* At_RK and *C*. *elegans*. **(i)** Larva of *C. elegans* captured after 24 h of interaction (scale bar = 20 μm), **(ii)** fungal colonization outside nematode body, traps adhered to cephalic and tail regions of *C. elegans* 3 days after predation (scale bar = 50 μm), **(iii)** magnification of area highlighted (panel ii) showing nematode captured by fungal adhesive trap (scale bar = 10 μm), **(iv,v)** fungus rupturing nematode cuticle (black arrows) (scale bar = 10 μm), **(vi)** fungus growing inside nematode body (white arrows) (scale bar = 20 μm).

Fungal parasitism against *M. incognita* J2s and *C. elegans* L3s was evaluated on water agar plates under *in vitro* conditions. The tested fungal isolates were found to be effective against both *M. incognita* and *C. elegans* after 3 days compared with control in the water agar plates (Supplementary Table 4). Results showed that *A. thaumasia* At_RK formed different types of traps to hunt the nematodes, *viz.*, 2D adhesive network, 3D adhesive network, and non-constricting rings (Figure 2). It caused approximately 82 ± 3.6% parasitism of *M. incognita* J2s. During parasitization, the fungal mycelium penetrated the nematode cuticle, developed inside its body, consumed the body contents, then ruptured the cuticle, and subsequently grew out of the body (Figure 3). The fungus mostly penetrated the nematode body along the lateral lines in both the nematodes (Figures 3, 4). Similarly, the percentage of *C. elegans* parasitized by *A. thaumasia* At_RK was approximately 73 ± 4.5%. It captured *C. elegans*, penetrated the cuticle and grew inside the body, and then ruptured the cuticle (Figure 4). It was noticed that it colonized approximately five to six *M. incognita* J2s but only one to two *C. elegans* L3s.

**FIGURE 5:**
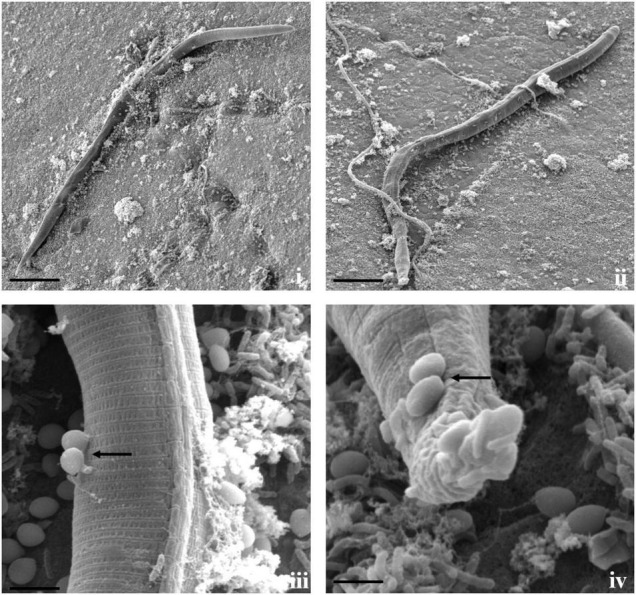
Scanning electron micrographs of interaction between *T. cylindrosporum* Tc_RK and *M. incognita*. **(i,ii)** Fungal colonization and parasitization on nematode cuticle (scale bar = 20 μm), **(iii,iv)** attachment of fungal spores onto nematode cuticle (black arrow) followed by spore germination (scale bar = 2 μm).

**FIGURE 6:**
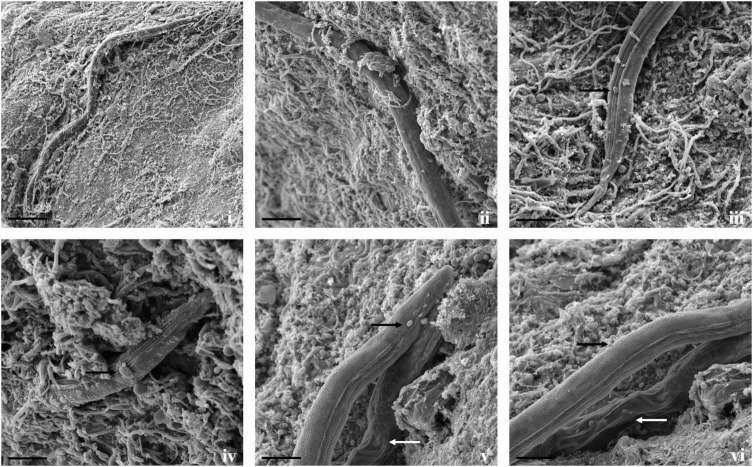
Scanning electron micrographs of interaction between *T. cylindrosporum* Tc_RK and *C. elegans*. **(i,ii)** Fungal colonization and parasitization on nematodes body (scale bar = 50, 20 μm), **(iii,iv)** attachment of fungal spores onto nematode cuticle (black arrow) (scale bar = 20 μm), **(v,vi)** comparison between fungus colonized nematodes having hypha inside its body (white arrows) and not colonized worms (black arrow) (scale bar = 20 μm).

Direct parasitism of *T. cylindrosporum* Tc_RK against both the nematodes was not as clear as in *A. thaumasia*. The capture and colonization of *M. incognita* by *T. cylindrosporum* were 65.2 ± 3.1% after 3 dpi and 57.7 ± 3.6% in *C. elegans* (Supplementary Table 4). The attachment of fungal spores on *M. incognita* cuticle was observed as a first step of the parasitism process (Figure 5). The fungal parasitization and spore attachment on *C. elegans* were significantly less compared with *M. incognita*, but consumption of nematode contents was detected quite well, as shown in Figure 6.

### *In vivo* Evaluation of Selected Fungi Against *M. incognita*

Fungal spore suspension having 1 × 10^6^ spores/ml was inoculated in the vicinity of tomato roots during transplantation. The selected fungal isolates significantly increased plant growth parameters in plant length, weight, and dry weight when compared with the control plants treated with only nematodes and SW. The maximum plant length (68.2 cm) and weight (14.92 g) were observed in the *T. cylindrosporum* Tc_RK-treated samples. Also, plants treated with *A. thaumasia* At_RK showed an average length of 66.8 cm, whereas the average weight was 13.4 g (Table 1). Interestingly, *A. thaumasia* At_RK-treated plants showed significantly higher dry weight (3.22 g) compared with only nematode-treated plants without the addition of fungal suspension.

**TABLE 1:** Evaluation of tested isolates on *M. incognita* infection and growth parameters of tomato at 45 dpi.

Treatments	Growth parameters			Infection parameters		
	Plant length (cm)	Plant wet weight (g)	Plant dry weight (g)	Number of galls	Number of egg masses	Number of eggs per egg mass
*Arthrobotrys thaumasia*	66.8 ± 8.01^b^	13.4 ± 2.3^b^	3.22 ± 0.36^b^	29 ± 16.2^b^	18.6 ± 6.6^c^	325.2 ± 31.1^c^
*Tolypocladium cylindrosporum*	68.2 ± 8.87^b^	14.92 ± 1.7^b^	3.04 ± 0.15^b^	43.4 ± 12.8^c^	7.2 ± 3.8^b^	221.2 ± 24.4^b^
Control Nematode	37.8 ± 3.57^a^	7.2 ± 0.8^a^	2.254 ± 0.27^a^	142 ± 5.4^d^	50.6 ± 4.5^d^	583.4 ± 40.7^d^
Healthy control	79.1 ± 6.34^c^	19.36 ± 0.82^c^	3.934 ± 0.56^c^	0^a^	0^a^	0^a^

*Each treatment had five replications. Values in same column followed by different letter(s) are significantly different at P ≤ 0.05 according to Duncan’s multiple range test.*

Furthermore, the effect of the selected fungi was determined on nematode disease burden in tomato plants inoculated with *M. incognita.* Results showed that the application of fungal suspension caused a significant reduction in nematode infection compared with control. It was found that average galling was in the range of 29 ± 16.2 in *A. thaumasia-* and 43.4 ± 12.8 in *T. cylindrosporum-*treated plants compared with control, which documented approximately 142 ± 5.4 that led to a decrease in galling intensity by 80 and 69%, respectively. Corroborating this, the number of egg masses was approximately 18.6 ± 6.6 and 7.2 ± 3.8 in *A. thaumasia-* and *T. cylindrosporum-*treated plants, respectively, compared with 50.6 ± 4.5 in control plants. Likewise, the number of eggs per egg mass was 325.2 ± 31.1 and 221.2 ± 24.4 in *A. thaumasia-* and *T. cylindrosporum-*treated plants, respectively, compared with 583.4 ± 40.7 in control plants. As a consequence, approximately 63 and 86% reduction in egg masses and 44 and 62% reduction in eggs per egg mass could be obtained in *A. thaumasia-* and *T. cylindrosporum*-treated plants, respectively. Ultimately, approximately 80 and 95% decline in nematode MF was observed in *A. thaumasia-* and *T. cylindrosporum*-treated plants, respectively (Table 1).

### Characterization of Volatile and Non-volatile Molecules of the Selected Isolates

Hexane soluble fraction of *A. thaumasia* and *T. cylindrosporum* was subjected to GC–MS analysis to identify VOCs. GC–MS analysis of hexane extract of *A. thaumasia* showed several peaks corresponding to 16 volatile compounds, representing 79.9% of the extract. The compounds are listed based on their retention time in Table 2. Interestingly, volatile sulfides such as *n*-propyl-butyl-disulfide (3.04%), bis (1-methylpropyl)-disulfide (2.80%), and dibutyl-disulfide (1.99%) were detected in the total ion chromatogram. 2-Methyl-1-pentanethiol (32.93%) was the major constituent followed by trimethyl-heptadien-4-one (23.34%), hexene-2,5-diol (5.08%), and chloro-3-butyltetrahydropyran (5.08%). Besides, dimethyl-pentanal (1.10%) and nerolic acid (1.10%) were also detected.

**TABLE 2:** Identification of volatile organic compounds (VOCs) of *A. thaumasia* and *T. cylindrosporum* using GC–MS analysis.

Compound Name	RT (min.)	Content (%)	Description
***A. thaumasia***			
Dimethyl-pentanal	2.17	1.1	–
2-Methyl-1-pentanethiol	2.18	32.93	It was used for preventing, destroying, or mitigating pests. It has a repellent insecticides odor (Matheny, 1980)
Ethylbutyl-hydroperoxide	2.28	0.1	It was found in *Moringa peregrine* leaves extracts as antimicrobial, anticancer, and antioxidant (Al-Owaisi et al., 2014)
Methylpentyl-hydroperoxide	2.34	0.42	
Trimethyl-heptadien-4-one	2.51	23.34	It was also found in *Artemisia lavandulaefolia* extracts works against *Plutella xylostella* and *Rhizoctonia solani* (Huang et al., 2018) It was found in *Malva sylvestris* extract works against *Haemonchus contortus* nematode (Al-Rubaye et al., 2017; Mravčáková et al., 2021)
Hexene-2,5-diol	2.74	5.08	It was isolated from *T. longibrachiatum*, having antibacterial activity against *Bacillus subtilis* (Sarsaiya et al., 2020)
Chloro-3-butyltetrahydropyran	2.85	5.08	–
Dodecadienal	3.1	0.8	It was found in *Ailanthus altissima* extracts, works against *Meloidogyne javanica* (Caboni et al., 2012)
Undecane	4.28	0.47	It was found in neem, *Azadirachta indica* leaf extract (Rady, 2018) It was found in *T. longibrachiatum* having antibacterial activity against *Bacillus subtilis* (Sarsaiya et al., 2020)
Tetramethyl-benzene	4.89	0.7	It was isolated from *T. harzianum* and *Alternaria alternate* PDB (Siddiquee et al., 2012; Encinas-Basurto et al., 2017)
Dibutyl-disulfide	5.71	1.99	It was found in *Ferula assa-foetida* oil, having antibacterial activity against *Streptococcus mutans*, *S. sanguis*, *S. salivarius*, *S. sobrinus*, and *Lactobacillus rhamnosus* (Daneshkazemi et al., 2019)
*n*-Propyl-butyl-disulfide	5.93	3.04	
Bis (1-methylpropyl)-disulfide	7.4	2.8	
Bicyclo-undeca-pentene	10.1	0.49	–
Ethyl-5-(ethylbutyl)-octadecane	10.22	0.49	It was found in *Salmali Niryasa* having antimicrobial activity against *Klebsiella pneumoniae*, *Staphylococcus aureus*, *E. coli*, and *Candida albicans* (Divya and Prabhu, 2015)
Nerolic acid	12.26	1.1	It was found in *Myrcia ovata* oils having antifungal activity against *Colletotrichum acutatum*, *Plenodomus destruens*, and *Thielaviopsis paradoxa* (White et al., 2019)
**Total**		79.93	
***T. cylindrosporum***			
Methyl-hexadecanol	2	2.08	It was found in *Satureja montana* oil having nematotoxic and phytotoxic effects (Faria et al., 2016)
Octadecenal	2.22	7.52	It was found as semiochemicals (attractants) such as pheromones, kairomones, and allomones that act to modify behavior of pests or their natural enemies It was identified as minor components in pheromone gland of female moths as part of the sex pheromone (Chen et al., 2018)
Ethenyloxy-octadecane	2.28	4.34	It was found in *T. longibrachiatum* having antibacterial activity against *Bacillus subtilis* (Sarsaiya et al., 2020)
Ethyl-3-methyl-benzene	2.54	8.1	It was identified from *T. harzianum* and *Alternaria alternate* PDB (Siddiquee et al., 2012; Encinas-Basurto et al., 2017)
Decane	2.81	17.7	It was found in *Capsicum annum* root extract, work against *Meloidogyne incognita* (Kihika et al., 2017) It was found in *Azadirachta indica* leaf extract (Rady, 2018)
Undecane	4.28	4.34	It is found as an active chemical compound of neem *Azadirachta indica* leaf extract (Rady, 2018)
Methylene-1-indene	6.65	0.94	It was found in many natural products and drug candidates with remarkable biological activities (Ye et al., 2010) It is used as a treatment of Alzheimer’s disease (Koca et al., 2016)
Dodecane	6.86	5.73	It was found in *Capsicum annum* root extract against *Meloidogyne incognita* (Kihika et al., 2017) It was isolated from *T. longibrachiatum*, having antibacterial activity against *Bacillus subtilis* (Sarsaiya et al., 2020) It was isolated from *Cymbopogon nardus* and *Dysphania ambrosioides*, did not exhibit nematicidal effects (de Freitas Silva et al., 2020)
Bis-(dimethyl-ethyl)-phenol	18.34	7.04	It was found in at least 169 species of bacteria and fungi having antibacterial, insecticidal, and nematicidal activities against *Caenorhabditis elegans* (Zhao et al., 2020)
Ethyl-5-(2-ethylbutyl)-octadecane	21.62	0.18	It was found in *Salmali niryasa* having antimicrobial activity against *Klebsiella pneumoniae*, *Staphylococcus aureus*, *E. coli*, and *Candida albicans* (Divya and Prabhu, 2015)
Hexadecanol	35.14	4.86	It was isolated from *Satureja montana* oil, which has nematotoxic and phytotoxic effects (Faria et al., 2016)
**Total**		62.83	

Likewise, GC–MS analysis of hexane extract of *T. cylindrosporum* also displayed various major and minor peaks corresponding to 11 VOCs. Compounds are listed as per their elution from the HP-5MS column (Table 2). Most of the peaks of the total ion chromatogram of the sample were identified as hydrocarbons and alcohols. Among these, decane (17.70%) was the most abundant. Other major compounds identified were ethyl-3-methyl-benzene (8.10%), octadecenal (7.52%), bis-(dimethyl-ethyl)-phenol (7.04%), dodecane (5.73%), hexadecanol (4.86%), and undecane (4.34%).

Furthermore, UPLC-QToF-ESI-MS analysis of a methanolic extract of *A. thaumasia* revealed various peaks in the total ion chromatogram; however, six metabolites were identified tentatively based on their molecular ion peaks, considering error mass (δ) value within 10 ppm (Table 3). These six metabolites were cyclo(L-Pro-L-Val) or piperazinone (1), cyclo(L-Pro-L-Leu), a pyrazine-1,4-dione derivative (2), paganin A (3), talathermophilin E (4), dactylarin (5), and trichodepsipeptide A (6). Among these, cyclo(L-Pro-L-Leu) is a homodetic cyclic peptide consisting of leucyl and prolyl amino acid residues. Furthermore, these metabolites were also confirmed from their possible fragmentation pattern in high-resolution MS. Cyclo(L-Pro-L-Val) or piperazinone was identified from its molecular ion peak at m/z 196.1201. Besides, fragmentation of the molecular ion peak resulted in daughter ion peaks at m/z 154 and m/z 126, originated due to sequential loss of isopropyl (43 amu) and carbonyl (28 amu) moieties. Another metabolite, cyclo(L-Pro-L-Leu), showed a characteristic molecular ion peak at m/z 210.1373, which on fragmentation produced possible daughter ions at m/z 195, 166, and 138, originated due to subsequent breaking of methyl (15 amu), ethyl (29 amu), and carbonyl groups (Supplementary Figure 4A). Similarly, the other four metabolites, paganin A, talathermophilin E, dactylarin, and trichodepsipeptide A, were characterized from their respective molecular ion, sodiated or adduct ion peaks at m/z 152.0940, 312.1711, 327.0921, and 711.4034.

**TABLE 3:** Identification of metabolites in *A. thaumasia* and *T. cylindrosporum* using UPLC-QToF-ESI-MS analysis.

Metabolites (tentative)	Molecular formula	Neutral mass (Da)	Observed m/z	Error mass (ppm)	Adduct	Description
***A. thaumasia***						
Cyclo(l-Pro-l-Val)	C_10_H_16_N_2_O_2_	196.1212	196.1201	1.53	M^+^	It was isolated from different fungi and bacteria as a secondary metabolite (Capon et al., 2007) It was isolated from entomopathogenic fungus *Cordyceps sinensis* (Yang et al., 2011)
Cyclo(l-Pro-l-Leu)	C_11_H_18_N_2_O_2_	210.1368	210.1373	2.38	M^+^	It was produced by *Achromobacter xylosoxidans*, a bacterium that inhibits Aflatoxin production by *Aspergillus parasiticus* (Yan et al., 2004) It has been isolated from various bacterial and fungal species, including *Streptomyces* (Li et al., 2006)
Paganin A	C_9_H_11_O_2_	151.0858	152.0940	−3.51	H^+^	It was found in *Arthrobotrys entomopa*, enhance adhesive knobs formation (Degenkolb and Vilcinskas, 2016)
Talathermophilin E	C_18_H_21_N_3_O_2_	311.1634	312.1711	−3.86	H^+^	It was found in *Talaromyces thermophilus*, having a nematicidal activity (Guo et al., 2011)
Dactylarin	C_16_H_16_O_6_	304.0947	327.0921	−8.54	Na^+^	It was isolated from *Dactylaria lutea*, having antiprotozoal activity against *Leishmania braziliensis* and *Entamoeba invadens* (Kettner et al., 1973) It was found in *Alternaria* PDB culture (Zheng et al., 2012)
Trichodepsipeptide A	C_36_H_56_N_4_O_9_	688.4047	711.4034	−1.19	Na^+^	It was isolated from *Trichothecium* sp., showing weak antibacterial activity on *Staphylococcus epidermids* and *Enterococcus duran* (Amagata et al., 2006; Sy-Cordero et al., 2011) It was isolated from many fungi *viz., Acremonium*, *Alternaria*, *Aspergillus*, *Beauveria*, *Fusarium*, *Isaria*, *Metarhizium*, *Penicillium*, and *Rosellina*. Having various biological activities such as antimicrobial and insecticidal (Wang B.L. et al., 2018, Wang X. et al., 2018)
***T. cylindrosporum***						
Tolypocladenols A1/A2	C_21_H_25_NO_4_	355.1784	356.1852	−5.35	H^+^	They were isolated from *Tolypocladium cylindrosporum*, inhabits lichen *Lethariella zahlbruckneri* (Li et al., 2015) They were cytotoxic to human cancer cells (Kebede et al., 2017) They were isolated from *Acremonium* spp. and *Trichoderma hamatum* (Romano et al., 2018)
Tolypyridone A	C_21_H_25_NO_3_	339.4350	363.4416	−6.19	H^+^ + Na^+^	
Tolypyridone B	C_15_H_21_NO_2_	247.3380	248.3480	5.24	H^+^	
Pyridoxatin	C_15_H_21_NO_3_	263.1521	526.3010	−6.08	dimer	
Penicillenol B1/B2	C_16_H_25_NO_3_	279.1834	280.1916	−1.79	H^+^	They were identified from *Penicillium* spp. associated with *Aegiceras corniculatum* (Lin et al., 2008) They were isolated from *Aspergillus restrictus*, inhibited biofilm formation of pathogenic *Candida albicans* fungus (Wang X. et al., 2017)
Terpendole E	C_28_H_39_NO_3_	437.2930	460.2962	7.31	Na^+^	It was isolated from *Neotyphodium coenophialum* endophytic fungus, which decreased population of plant-parasitic nematodes (Takach and Young, 2014; Rogers et al., 2016)
Destruxin A	C_29_H_47_N_5_O_7_	577.3475	578.3556	−1.03	H^+^	It was isolated from entomopathogenic fungus, *Metarhizium anisopliae* which has broad-spectrum insecticidal effects (Liu and Tzeng, 2012; Shakeel et al., 2018; Wang et al., 2020)
4-Chloro-2-phenylphenol	C_12_H_9_ClO	204.0342	205.0446	8.33	H^+^	It was isolated as a plant secondary metabolite having nematicidal activity against *M. incognita* (Ohri and Pannu, 2010)
Acetamido-6-[(O-methyl-glucopyranosyl) oxy]anthraquinone	C_23_H_24_NO_9_	458.1442	458.1451 481.1372	1.96	M^+^ H^+^ + Na^+^	It was isolated from *Photorhabdus* spp. (symbiotic bacteria of entomopathogenic nematode) having an antimicrobial and mosquitocidal activity (Joyce et al., 2011; Ahn et al., 2013)

UPLC-QToF-ESI-MS analysis of a methanolic extract of *T. cylindrosporum* tentatively revealed the identification of a total of nine metabolites. These metabolites, tolypocladenols A1/A2 (i), tolypyridone A (ii), tolypyridone B (iii), penicillenol B1/B2 (iv), pyridoxatin (v), terpendole E (vi), destruxin A (vii), 4-chloro-2-phenylphenol (viii), and acetamido-6-[(O-methyl-glucopyranosyl)oxy]anthraquinone (ix), were characterized based on their exact monoisotopic mass value (error mass < 10 ppm) and mass fragmentation pattern (Table 3). The first metabolite, tolypocladenols A1 or A2, was identified from its adduct ion peak at m/z 356.1852. Fragmentation of the adduct ion produced daughter ion peaks at m/z 327 and 191 originated due to the removal of two methyl (−28 amu) and side hydrocarbon chains (136 amu) (Supplementary Figure 4B). Similarly, the remaining eight metabolites were also characterized from their corresponding fragmentation pattern of the molecular ion peaks.

## Discussion

RKNs, *Meloidogyne* spp., are the most damaging endoparasites with a wide range of hosts resulting in huge losses in various crops worldwide. Climatic fluctuations have resulted in the evolution of new races of nematodes that overcome the previous source of resistance and cause diseases. To date, it has been very difficult to recommend a promising nematode management tool that is effective, environmentally safe, economical, and harmless to the non-targets. In this regard, biocontrol agents have been preferred due to their potential against target parasitic nematodes. NTFs hold great potential as biocontrol agents, as they capture nematodes by producing trapping devices from their vegetative mycelia and also produce various metabolites as nematicidal weapons that have antagonistic activity against infective juveniles (Wang X. et al., 2018; Kuo et al., 2020). To understand its importance in the integrated pest management programs and to enrich the options of the fungi to be used as biocontrol agents, the present study was undertaken to characterize nematophagous fungal isolates of *Arthrobotrys* and *Tolypocladium*, which were isolated using *C. elegans* and *M. incognita* as bait (Kassam et al., 2021). These two isolates were used for species identification and evaluation against *C. elegans* and *M. incognita*.

For morphological characterization, structure, nature, and color of the fungal colonies, along with the measurement of taxonomic features such as size and shape of conidia, conidiophores, and phialides, as well as the presence of chlamydospores, were considered. In the case of isolate *Arthrobotrys*, the conidia were inverted pear-shaped, 1–3 septate, and 24.58–60 × 10.15–22.88 μm in size. Furthermore, the trapping device is not a constricting ring but an adhesive network that matches the description of *A. thaumasia* (Wang X. et al., 2017; Zhang et al., 2021). Thus, both the morphological characteristics and nematophagous behavior of our isolate were in conformity with the previous description of *A. thaumasia* that was designated as *A. thaumasia* At_RK. It is important to reiterate that this was isolated from dead *M. incognita*, unlike the other geographical strains reported (Kuo et al., 2020). This is the first report of the presence of *A. thaumasia* in India. In the case of *Tolypocladium* isolate, conidia were hyaline, smooth-walled, short cylindrical, straight, or slightly curved, both ends obtusely rounded, and one-celled 2–4.3 × 1.3–1.7 μm, adhering to the phialide tips in slimy heads. Phialides were 4.5–8.5 × 2–3.2 μm in size and consisted of an inflated ellipsoidal to cylindrical base tapering abruptly to a thin neck, which often gives a bent appearance. All these features corresponded with the description of *T. cylindrosporum* (Bissett, 1983; Samson and Soares, 1984), and hence, we designated our isolate as *T. cylindrosporum* Tc_RK, which is also the first report from India. It is also important to emphasize that this isolate was from the dead nematodes, unlike the isolate used for comparison, which was from insects (Samson and Soares, 1984).

Morphological identification of the selected fungi was further supported by molecular characterization using two molecular markers. In the case of ITS, the sequence *A. thaumasia* At_RK showed a high similarity to the already reported strain of *A. thaumasia* available in GenBank. Likewise, the sequence of *T. cylindrosporum* Tc_RK also showed maximum identity to the already reported strain of *T. cylindrosporum*. These findings confirmed the identity and presence of *A. thaumasia* and *T. cylindrosporum* in the Indian rhizospheric soils. Our results showed that analysis of molecular variation and maximum composite likelihood analysis using ITS and β-tubulin markers revealed a considerable degree of differentiation between geographical isolates. The results also demonstrate that the ITS and β-tubulin markers are useful for phylogenetic analysis and classification of *Arthrobotrys* and *Tolypocladium* species. Li et al. (2005) also studied phylogenies of NTFs deduced from sequence analyses of 5.8S rDNA, 28S rDNA, and β-tubulin genes and redefined the systematic classification of nematophagous fungi and amended the generic analysis of NTF based on types of trapping devices.

Furthermore, these isolates were studied under different media, incubation temperatures, and pH levels to analyze the growth rates and sporulation characteristics. The SNA media having pH 9 at 30°C showed the best growth rate, whereas RBA media supported high sporulation in the case of *A. thaumasia* At_RK. The results reported herein are in line with those of Wang F.H. et al. (2017), who obtained the highest growth at the optimal temperature at 30°C for the same fungus. In contrast, Fernandez et al. (1999) showed that *A. oligospora* exhibited the best growth rate at 20°C, and another fungus, *Duddingtonia flagrans*, grew at 10°C and formed trapping nets more slowly at this temperature when induced by nematodes. The Indian isolate of *A. thaumasia* At_RK that grew at optimum temperature at 30°C could be advantageous as a biological control agent in subtropical environments that remain at 30°C for a longer period. Additionally, the results of the current study match with the findings of Wang F.H. et al. (2017), who reported optimum growth of *A. thaumasia* on a media having pH 9 and 10. Contrastingly, there are no previous reports about the impact of different media, incubation temperatures, and pH levels on *T. cylindrosporum* growth and sporulation; however, the present study showed the best growth rate of *T. cylindrosporum* Tc_RK in the PDA media, having pH 6 at 20°C, whereas SNA medium supported high sporulation level. These optimized conditions for getting maximum fungal sporulation and growth could be highly useful for the large-scale production of these fungi in the future.

Most importantly, the utility of both fungi was primarily demonstrated by their ability to parasitize *M. incognita* and *C. elegans* under *in vitro* conditions. The selected fungal isolates were found to be effective against both the assessed nematodes after 3 days compared with control. Our results revealed that *A. thaumasia* showed significantly higher parasitism compared with *T. cylindrosporum* and water agar control plates (*p* < 0.01). Direct parasitism of the Indian isolate of *A. thaumasia* At_RK against *M. incognita* was comparatively higher (82%) than the efficacy obtained with the Korean isolate of *A. thaumasia* Nema-1 (55%) (Park et al., 2011). The finer details such as different types of traps and penetration in the region of lateral lines, etc., during *A. thaumasia* parasitizing *M. incognita* J2s was lacking, and the same has been documented for the first time in the present study.

The culture filtrate of *T. cylindrosporum* Tc_RK provided 87.3% mortality of *M. incognita* compared with control. So far, to the best of our knowledge, this is the first report about the ability of *T. cylindrosporum* to parasitize *M. incognita.* Interestingly, the results revealed that mortality of *C. elegans* caused by these two local fungal isolates was comparatively less, although *A. thaumasia* At_RK was isolated from dead *C. elegans*. The possible reason underlying lower mortality of *C. elegans* may be due to the fast movement that could have prevented them from immobilization and paralyzation, and/or body secretion of *M. incognita* could have attracted the trapping fungi relatively more.

During the *in vivo* evaluation of the isolated fungi against *M. incognita-*infected tomato, there was a significant increase in plant growth parameters compared with control plants infected with only nematodes. The tested isolates did not show any promotion in growth parameters compared with healthy control, but they enhanced plant growth in nematode-infected plants as a sign of protection provided against them. Further application of fungal filtrate of *A. thaumasia* At_RK caused a significant reduction in nematode disease burden per plant compared with control. Similar observations have been recorded by Park et al. (2012) using the culture filtrate of *A. thaumasia* on *M. incognita.* Likewise, the application of fungal suspensions of *T. cylindrosporum* Tc_RK in the present study caused a significant reduction in the number of egg masses and eggs per egg mass. This ultimately led to a decline in the nematode MF up to 94.5%. This is the first report on the effect of *T. cylindrosporum* Tc_RK on nematode fecundity.

The successful nematode mortality brought about by both the tested nematophagous fungi in the present study led us to analyze their metabolite profiles, particularly the volatile and non-volatile chemical compounds (VOCs) using GC–MS and UPLC-QToF-ESI-MS to identify the metabolites responsible for the nematicidal activity. The results showed that *A. thaumasia* At_RK secreted both volatile and non-volatile compounds that could be responsible for nematicides. Two volatile compounds, 2-methyl-1-pentanethiol and trimethyl-heptadien-4-one, were identified at higher concentrations in this isolate. The activity of these compounds as a repellent, odor character, insecticides, and nematicides have already been reported earlier by Huang et al. (2018) and Mravčáková et al. (2021). Similarly, the compounds, dodecadienal, undecane, and nerolic acid observed throughout metabolite profiles in the present study have already been reported as nematicidal, antibacterial, and antifungal compounds (Caboni et al., 2012; White et al., 2019; Sarsaiya et al., 2020). Correspondingly, *A. thaumasia* At_RK secreted non-volatile compounds such as aganing, talathermophilin, and dactylarin found in other nematophagous fungi, *A. entomopa*, *Talaromyces thermophilus*, and *Dactylaria lutea* (Kettner et al., 1973; Degenkolb and Vilcinskas, 2016; Wang X. et al., 2017). Interestingly, none of these compounds were detected during the analysis of 100 isolates belonging to three species *A. oligospora, A. thaumasia*, and *A. musiformis*, during their interaction with *C. elegans* (Kuo et al., 2020). Thus, this is the first general metabolite profiling for *A. thaumasia* isolated from dead *M. incognita*, and hence, it could be promising for commercial exploitation in the future.

Likewise, our study with the Indian isolate of *T. cylindrosporum* Tc_RK showed secretion of volatile compounds, namely, methyl-hexadecanol, hexadecanol, decane, dodecane, and bis-(dimethyl-ethyl)-phenol, and the secreted compounds were reported to have antagonistic activity against nematodes (Faria et al., 2016; Kihika et al., 2017; Zhao et al., 2020). Other chemical compounds having nematicidal activities such as ethyl-3-methyl-benzene and undecane were also observed in the Indian isolate of *T. cylindrosporum* Tc_RK. These compounds were described to have nematicidal activities in the culture filtrate of *Trichoderma harzianum* and leaf extracts of *Azadirachta indica* (Encinas-Basurto et al., 2017; Rady, 2018). On the other hand, *T. cylindrosporum* Tc_RK also secreted non-volatile compounds with activity against nematodes, for instance, tolypocladenols, tolypyridone (A&B), and pyridoxatin, which were reported as metabolites from endolichenic isolate of *T. cylindrosporum*, *Acremonium* spp., and *Trichoderma hamatum* (Li et al., 2015; Romano et al., 2018). In addition to that, the tested isolate in the present study also secreted compounds such as terpendole E, 4-chloro-2-phenylphenol, destruxin A, and acetamido-6-anthraquinone, which were reported to have nematicidal activity (Ohri and Pannu, 2010; Ahn et al., 2013; Rogers et al., 2016; Shakeel et al., 2018; Wang et al., 2020) and not known to be present in *T. cylindrosporum*. It is also important to mention here that *T. cylindrosporum* Tc_RK isolated from dead *C. elegans* could be promising for nematode management due to its secretion of various novel metabolites with nematicidal properties.

## Conclusion

Despite several reports being available for the efficacy of nematophagous fungi as biocontrol agents, the present investigation established an in-depth study on the Indian isolates of *A. thaumasia* and *T. cylindrosporum* for RKN management. Both these fungi are reported for the first time from India. Furthermore, this is the first report showing the potential of *T. cylindrosporum* for RKN management. Besides, this is also an established report revealing the presence of nematicidal compounds in both fungi using metabolite profiling. In view of the potential demonstrated for both the selected fungi against *M. incognita*, they can be further explored for commercial product development.

## Data Availability Statement

The datasets presented in this study can be found in online repositories. The names of the repository/repositories and accession number(s) can be found in the article/Supplementary Material.

## Author Contributions

UR conceived, designed, and supervised the experiments. AH and RK analyzed all the experimental data and wrote the original draft of the manuscript. AK analyzed GC–MS and UPLC-QToF-ESI-MS data. UR and AH revised and edited the manuscript. All authors conducted the experiments, read, and approved the final manuscript.

## Conflict of Interest

The authors declare that the research was conducted in the absence of any commercial or financial relationships that could be construed as a potential conflict of interest.

## Publisher’s Note

All claims expressed in this article are solely those of the authors and do not necessarily represent those of their affiliated organizations, or those of the publisher, the editors and the reviewers. Any product that may be evaluated in this article, or claim that may be made by its manufacturer, is not guaranteed or endorsed by the publisher.

## Footnotes

1
https://www.ncbi.nlm.nih.gov/
