# Supplementary material for: Identification, Characterization, and Evaluation of Nematophagous Fungal Species of Arthrobotrys and Tolypocladium for the Management of Meloidogyne incognita
Source: Front Microbiol. 2021 Dec 10;12:790223. doi: 10.3389/fmicb.2021.790223 (PMC8702965; doi:10.3389/fmicb.2021.790223)
Supplement: Supplementary file 1 [file Data_Sheet_1.doc]

Supplementary Material

***Journal: Frontiers in Microbiology***

**Identification, characterization and evaluation of nematophagous fungal species of *Arthrobotrys* and *Tolypocladium* for the management of *Meloidogyne incognita***

**Rami Kassam^1^, Jyoti Yadav^1^, Gautam Chawla^1^, Aditi Kundu^2^, Alkesh Hada^1^, Nisha Jaiswal^1^, Haritha Bollinedi^3^, Deeba Kamil^4^, Prameela Devi^4^ and Uma Rao^1*^**

^1^Division of Nematology, ICAR-Indian Agricultural Research Institute, New Delhi, India

^2^Division of Agricultural Chemicals, ICAR-Indian Agricultural Research Institute, New Delhi, India

^3^Division of Genetics, ICAR-Indian Agricultural Research Institute, New Delhi, India

^4^Division of Plant Pathology, ICAR-Indian Agricultural Research Institute, New Delhi, India

* Correspondence to:

Uma Rao,

Division of Nematology,

ICAR-Indian Agricultural Research Institute, New Delhi - 110 012, India

E-mail: umarao@iari.res.in

Telephone Numbers: 011-25846626, 25842721, 25846400, 27550355

Fax Number: 011-25846626

ORCiD: https://orcid.org/0000-0002-1233-2921


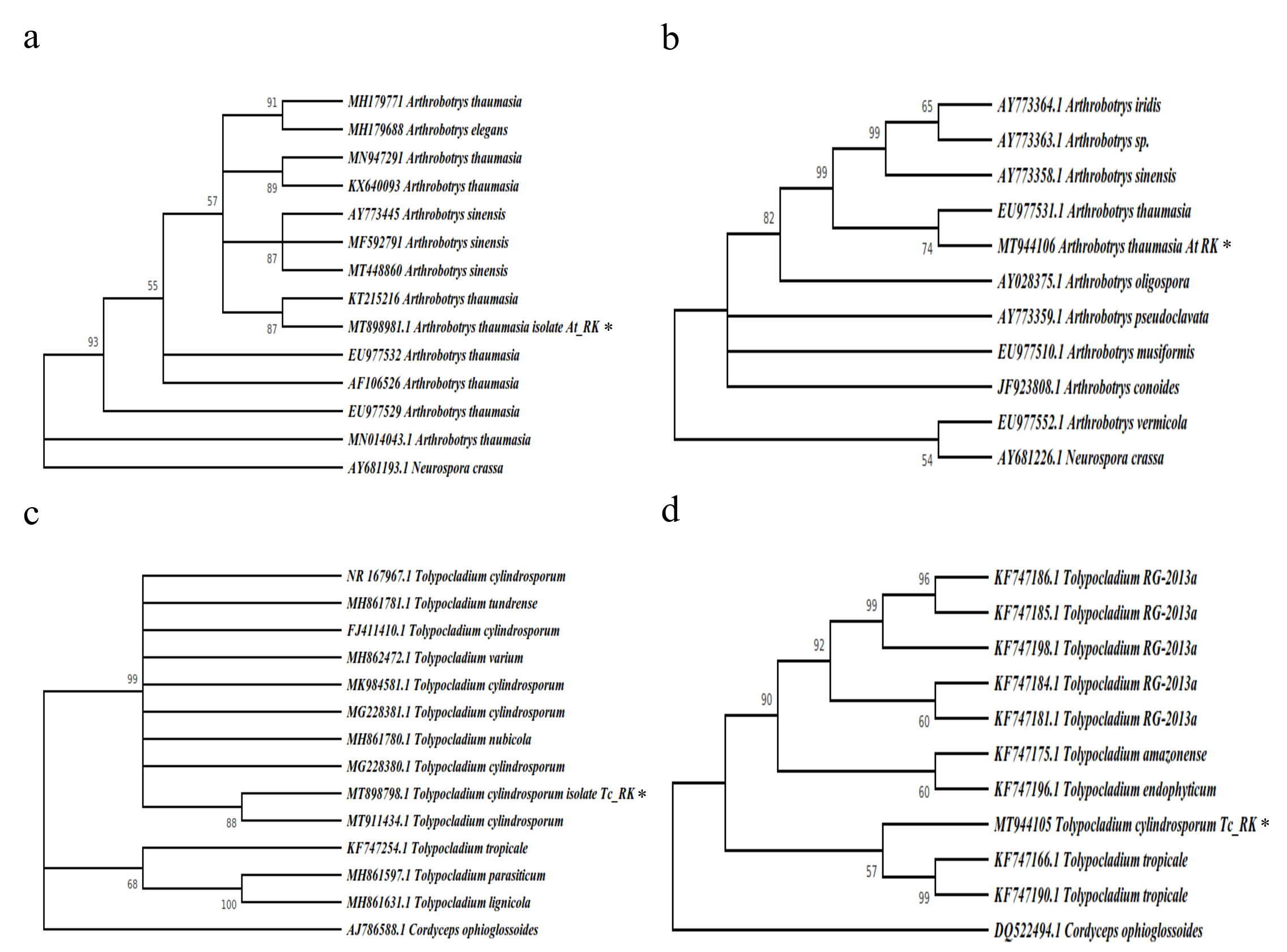


**FIGURE S1** Phylogenetic tree based on confidently aligned ITS and β–tubulin sequences using Maximum Composite Likelihood (MCL) method. The numbers on the tree branches indicate the bootstrap values from 1000 replicates using Mega-X. **a** *A. thaumasia* using ITS marker, **b** *A. thaumasia* using β–tubulin marker, **c** *T. cylindrosporum* using ITS marker, **d** *T. cylindrosporum* using β–tubulin marker. Isolates in the present study are shown with asterisks


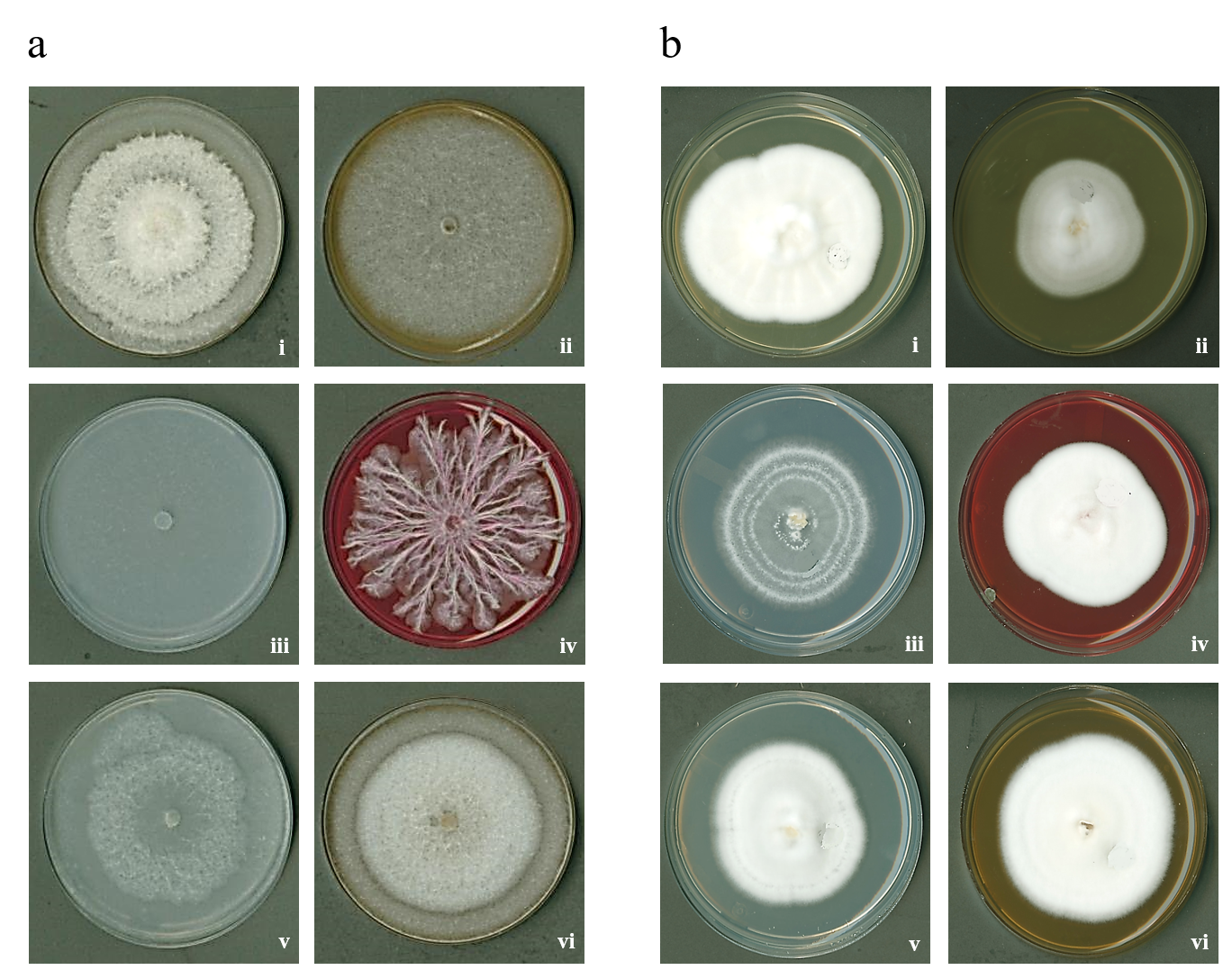


**FIGURE S2** Colony growth of **a** *A. thaumasia* At_RK, and **b** *T. cylindrosporum* Tc_RK on different media 10 days after inoculation at 25°C. (**i**) PDA, (**ii**) CMA, (**iii**) SNA, (**iv**) RBA, (**v**) PYG, and (**vi**) CzMA


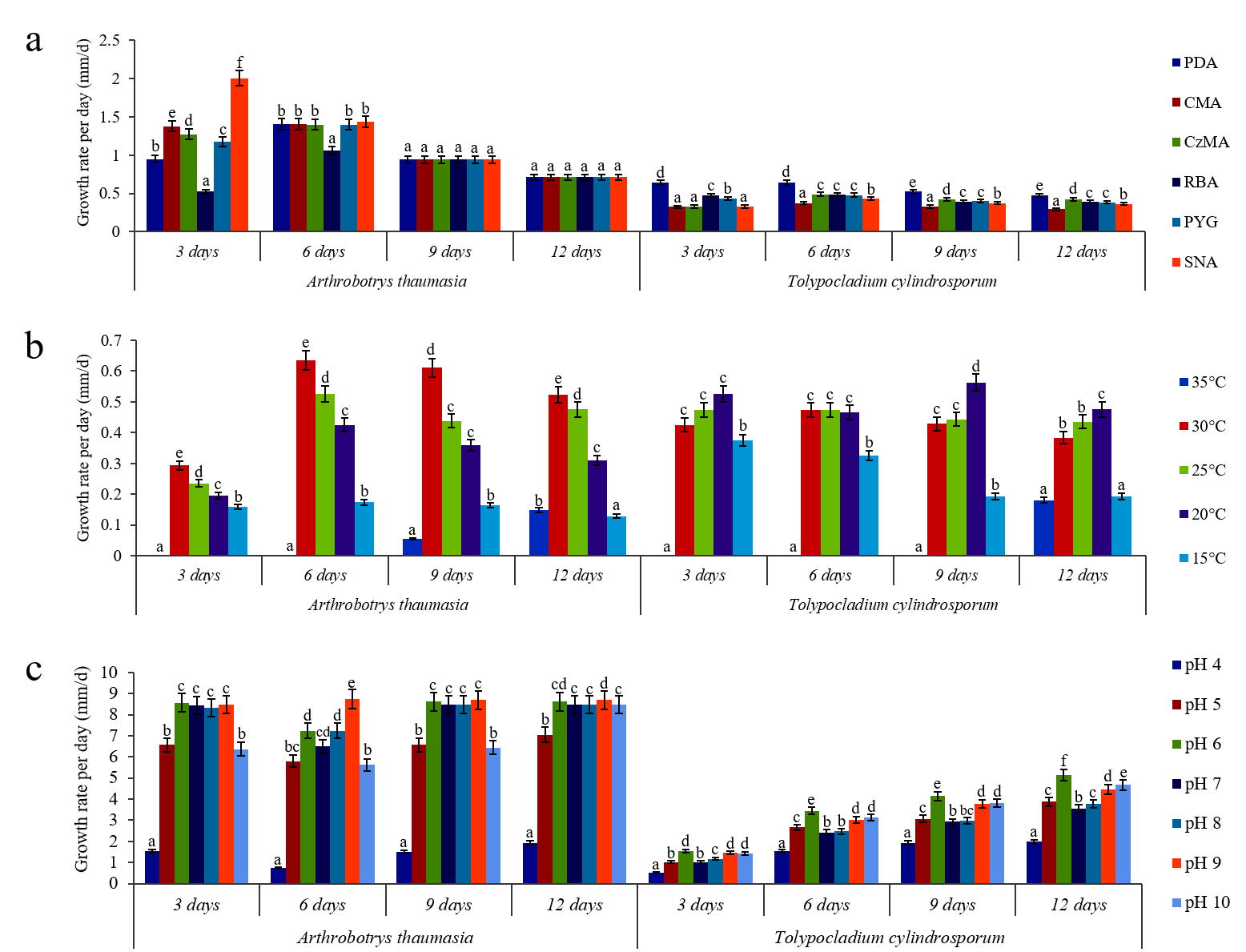


**FIGURE S3** Effect of various factors on growth rates of *A. thaumasia* and *T. cylindrosporum* at 3, 6, 9 and 12 days after inoculation. **a** different media (PDA, CMA, CzMa, RBA, PYG and SNA), **b** different incubation temperatures (15, 20, 25, 30 and 35°C) **c** different pH levels (4-10)


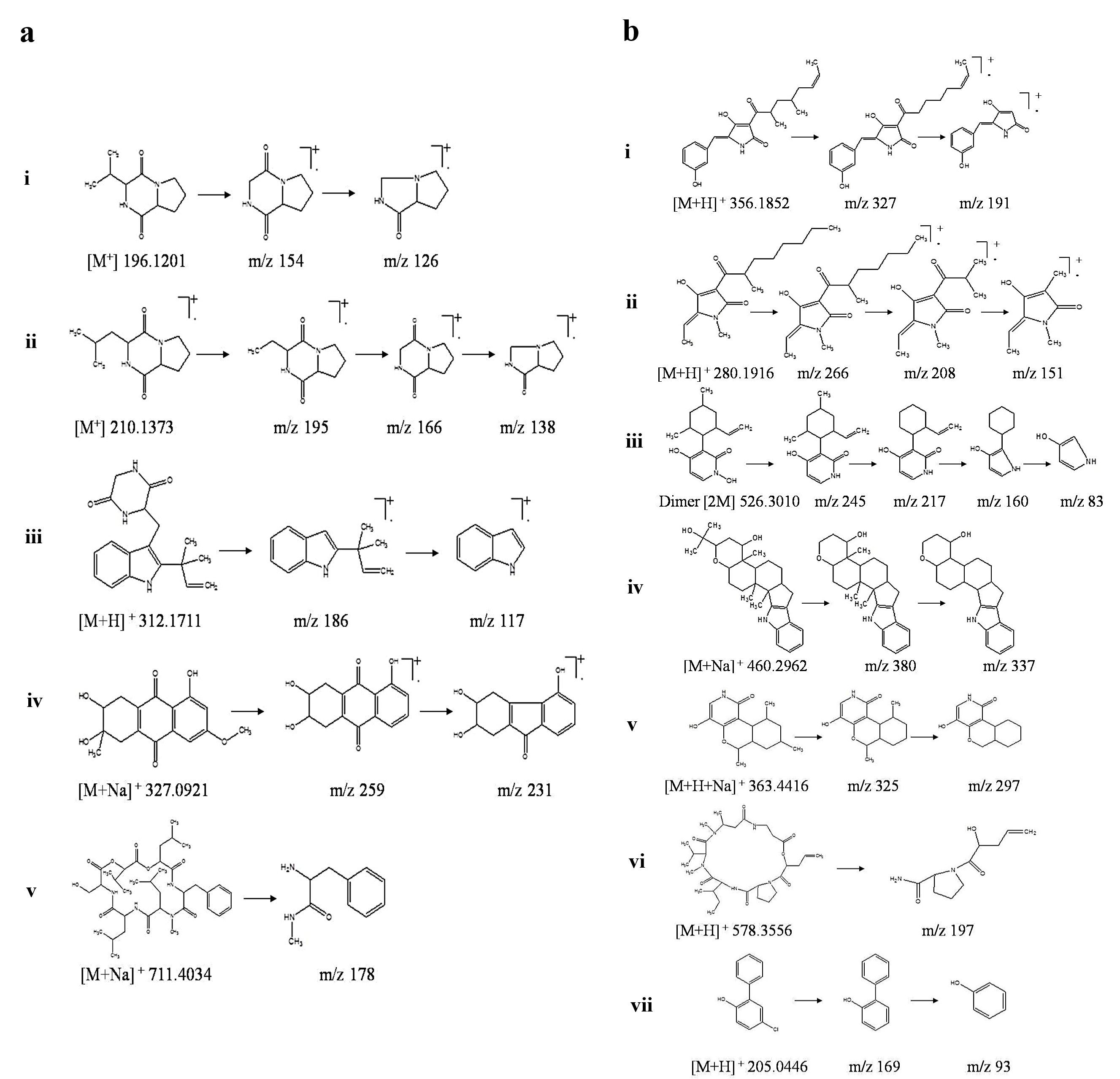


**FIGURE S4** Structure of major metabolites extracted from the tested fungi **a** *A. thaumasia* At_RK, (**i**) Cyclo(l-Pro-l-Val), (**ii**) Cyclo(l-Pro-l-Leu), (**iii)** Talathermophilin E, (**iv**) Dactylarin, (**v**) Trichodepsipeptide A, **b** *T. cylindrosporum* Tc_RK, (**i**) Tolypocladenols, (**ii**) Penicillenol, (**iii**) Pyridoxatin, (**iv**) Terpendole, (**v**) Tolypyridone A, (**vi**) Destruxin A, (**vii**) 4-Chloro-2-phenylphenol

**Table S1** Morphological characteristics of the tested fungal colonies and their morphometric characteristics used for identification

| **Character** | **Fungi species** | |
| --- | --- | --- |
|  | ***Tolypocladium cylindrosporum*** | ***Arthrobotrys thaumasia*** |
| Colony morphology on PDA | Colonies effuse hairy, whitish cream.  Revers yellow to pale, no exudate | Colonies straw white with raised concentric rings, thin and hairy.  Reverse pale to yellowish brown |
| Colony morphology on CMA | Colonies restricted in growth, whitish gray, thin walled.  Reverse yellow, no exudate | Colonies restricted and starving, dust white in color.  Reverse pale |
| Colony morphology on CzMA | Colonies effuse fluffy, white in color.  Reverse brown, no exudate | Colonies white in cream in color with consented rings, thin and hairy.  Reverse pale to brown |
| Colony morphology on RBA | Colonies fluffy, effuse hairy, pure white.  Revers orange to pink to salmon | Colonies distinct hairy, pink and thick thread like pattern.  Reverse pale |
| Colony morphology on PYG | Colonies raised, effuse, slow in growth, hairy.  Reverse pale to lemon yellow, no exudate | Colonies dull white in color, thin and starving.  Reverse pale |
| Colony morphology on SNA | Colonies less mycelium, with consented rings, restricted in growth. Reverse pale to cream, no exudate | Colonies very degenerated in mycelia growth, white in color.  Reverse pale |
| Conidium length (μm) | 2–4.3 | 24.58–60 |
| Conidium width (μm) | 1.3–1.7 | 10.15–22.88 |
| Conidium shape | Hyaline, smooth walled, short cylindrical, straight or slightly curved, both ends obtusely rounded | inverted pear shape, 1–3 septa |
| Conidiophores length (μm) | 31–44 | 211–446 |
| Conidiophores width (μm) | 1.1–2.8 | 2.3–5.4 |
| Phialide length (μm) | 4.5–8.5 | – |
| Phialide width (μm) | 2–3.2 | – |
| Chlamydospores diameter (μm) | absent | present |

**Table S2** Isolation and identification of nematophagous fungi from Indian soils using *Caenorhabditis elegans* and *Meloidogyne incognita* as bait

| **State** | **Plant species (Botanical name; Family)** | **Fungi  Species** | **Used Bait** | **Isolate code** | **Latitude (N)** | **Longitude (E)** | **GenBank accession No.** | | **ITCC accession No.** |
| --- | --- | --- | --- | --- | --- | --- | --- | --- | --- |
|  |  |  |  |  |  |  | **ITS** | **β-tubulin** |  |
| Jammu and Kashmir | Grass (*Saccharum spontaneum*; Poaceae) | *Tolypocladium cylindrosporum* | *C. elegans* | Tc_RK | 33°94'32.62" | 74°38'50.24" | MT898798 | MT944105 | ITCC8969 |
| Delhi | Rose (*Rosa sinensis*; Rosaceae) | *Arthrobotrys thaumasia* | *M. incognita* | At_RK | 28°36'33.07" | 77°11'11.91" | MT898981 | MT944106 | ITCC8970 |

**Table S3** Effect of different media on sporulation of the tested fungal isolates

| **Media** | ***Arthrobotrys thaumasia***  **Sporulation (spore/ml)** | ***Tolypocladium cylindrosporum***  **Sporulation (spore/ml)** |
| --- | --- | --- |
| PDA | 5.3 × 10^4^ | 7203.3 × 10^4^ |
| CMA | 3.4 × 10^4^ | 3427.2 × 10^4^ |
| CzMA | 9.1 × 10^4^ | 112.7 × 10^4^ |
| RBA | 30.3 × 10^4^ | 72.1 × 10^4^ |
| PYG | 7.7 × 10^4^ | 3179.4 × 10^4^ |
| SNA | 6.3 × 10^4^ | 9006.3 × 10^4^ |

**Table S4** Effect of the tested fungi on parasitization and mortality of *M. incognita* J2s (*Mi*) and *C. elegans* L3 (*Ce*)

under *in* *vitro* conditions

| **Treatments** | **Culture filtrates** | | **Water agar plates** | |
| --- | --- | --- | --- | --- |
|  | ***Mi* mortality J2 %** | ***Ce* mortality L3 %** | ***Mi* parasitized J2 %** | ***Ce* parasitized L3 %** |
| *Arthrobotrys thaumasia* | 57.7 ± 3.5 ^b^ | 53.7 ± 2.3 ^b^ | 82 ± 3.6 ^c^ | 73 ± 4.5 ^c^ |
| *Tolypocladium cylindrosporum* | 87.3 ± 6.02 ^c^ | 64 ± 3.6 ^c^ | 65.2 ± 3.1 ^b^ | 57.7 ± 3.6 ^b^ |
| Control Nematode | 0 ^a^ | 0 ^a^ | 0 ^a^ | 0 ^a^ |

Each treatment had five replications. Values in the same column followed by different letter(s) are significantly

different at *P ≤* 0.01 according to the Duncan’s multiple range test
